# “It is a good idea, but…” A qualitative study of implementation of ‘Individual Plan’ in Norwegian mental health care

**Published:** 2012-03-30

**Authors:** Lene Chr Holum

**Affiliations:** Centre for Child- and Adolescent Mental Health Eastern and Southern Norway (R-BUP) and Oslo University Hospital, Norway

**Keywords:** Implementation, management, integrated care model, mental health

## Abstract

**Aim of the study:**

The aim of the study is to explore and describe what hampers and promotes the implementation of ‘Individual Plan’—Norway’s answer to integrated care, and to discuss the findings according to implementation theory and research.

**Background:**

‘Individual Plan’ is a master-plan intended to increase user-participation and provide better coordination of measures for patients in need of extensive and long-term health-care services. Norwegian Health Authorities used a dissemination strategy to implement ‘Individual Plan’ but managers within health and social care could choose their own way of implementation in their organisation.

**Methodology:**

Twenty-two managers from different clinics and organisational levels within mental health care were interviewed with an in-depth semi-structured interview about the implementation process in their organisation. The analysis was primarily made according to systematic text condensation.

**Findings:**

The findings describe different implementation processes and how the managers identified with the usefulness of ‘Individual Plan’ as a tool, choice of practical implementation strategies, the manager’s own role, characteristics of organisational culture as well as how the manager considered external factors such as administration, lack of time and resources. The evolved implementation themes are discussed within a frame of existing knowledge and theory.

**Conclusion:**

A complex picture of barriers, dilemmas and benefits emerges, both internal and external to an organisation as well as at a personal level that need to be taken into consideration in forthcoming implementation processes to increase the rate of success.

## Introduction

To achieve well-implemented high quality care for persons with long-standing and complex mental health problems, the way we organize mental health service is of great importance. Internationally, there is increased focus on compensating for the fragmented delivery of care by promoting integrated care and collaboration, but the services still suffer from organisational, clinical and cultural fragmentation [1]. WHO defines integrated care as a concept bringing together inputs, delivery, management and organisation of services. Integration is a means to improving services in relation to access, quality, user satisfaction and efficiency. Norwegian health care has been searching for methods to deliver more coordinated help according to the patients’ needs, and ‘Individual Plan’ (hereafter IP) is a national answer to integrated care. To develop methods for integrated care is a challenge, but implementing them to function properly is an even bigger challenge [2]. This paper discusses some managers description and reflections on the implementation process within mental health specialist services.

### IP in relation to integrated care

IP was introduced into the Norwegian health legislation in 2001 because, in spite of great need of services, several patient groups were not offered acceptable measures. The purpose was to achieve a more long-term and unified mode of thought around patients (patients and users are used as synonyms in this paper) in need of extensive services [3]. IP is intended as a tool to ensure that services and measures work and are experienced as unified, coordinated, and individually adapted to each patient. The patient’s own goals govern the planning work [4], and there will be a personal coordinator. IP is a written master plan, intended to span all areas of service and levels of administration. It shall encompass other elements such as treatment plans, measure plans, education plans, etc., and the patient is supposed to have access to all services and measures needed. IP reflects the recent development in health and social care with an increasing focus on collaboration and user-involvement (i.e., The Ottawa Charter for health promotion [5]). Stimulating changes towards integrated care involves factors dealing with the service-providers, the patients, social contexts, as well as economic, administrative and organisational factors [6].

### Implementation and improvements of practice

The Norwegian Health Authorities initiated the implementation of IP with what the author understood as a dissemination strategy [7]. This strategy is not necessarily adequate implementation, which is understood as a more systematic use of strategies to introduce changes. Dissemination is only a targeted distribution of information, mainly written, but also through meetings, with the intent of spreading information about laws and regulations to a specific audience: health and social care workers on this occasion. Thereafter, each municipality, health region, hospital or manager decided more or less independently how they understood the regulations and instructions, and how they made IP work. Use of legal obligation, as with IP, is seen as one of the most powerful methods of influencing behavioural changes, but at the same time the long-term results of this approach are not clear [8]. The health authorities have neither implemented nor evaluated IP in a systematic way. A systematic strategy would have made an evaluation more effective and easier to accomplish [9–11]. There is a field of theory and research on implementation and innovation effectiveness [6, 10, 12–16], but most of the time implementation processes receive little attention [17], as with IP.

Implementation can be defined as the process of putting an innovation like IP into use [14]. Frequently used implementation theories cover the likelihood of a change to be adopted both at organisational and individual levels, and the premises for such a change to happen [14–16, 18]. “Organisational theory of implementation effectiveness” emphasises determinants of effective implementation to be an organisation’s readiness for change, quality of implementation policies and practices, climate, and congruency of innovation and values [14]. “Roger’s diffusion of innovation model” is mostly used at an individual level. It covers the process from receiver variables; personality, social characteristics and perceived need of innovation, through a process of knowledge and persuasion before a decision can be arrived at; an adoption or rejection of the innovation [15]. To facilitate any kind of innovation a practical implementation strategy is necessary, covering elements as directions, training, supervision, reminders, technical support, sufficient resources, local opinion leaders and leadership engagement [10, 13, 19]. To sum up the main elements of this implementation literature; the organisation, the individuals and the process itself will all play an important part in implementation and adoption of a new tool like IP.

### Aim of the study

The aim of the study is to describe and explore experiences and reflections on the process of implementation of IP, especially what hampers and promotes the process seen through the lenses of a number of managers within Norwegian mental health care. The findings are discussed within a frame of implementation theory since identifying personal and organisational barriers and dilemmas is essential in order to achieve a better understanding of the processes as well as to prepare for improved future strategies for implementing new tools and guidelines.

## Methodology and design

The present study on implementation is part of a larger qualitative project on how IP was implemented and used in a given population of youths with long-term mental health problems; hereunder what hampered or promoted the IP function as intended according to the interviewed managers, patients, relatives and coordinators. The whole project is mainly descriptive and evaluative, searching to gain an insight of how IP is put into action. According to Patton [20], evaluation research concerns studying what promotes or hampers the appropriate function of a method or a tool, like IP on this occasion. To provide an understanding of the processes carrying out the implementation of IP, concerning experiences and reflections, a qualitative approach with semi-structured in-depth interviews was deemed appropriate.

### Subjects and context

Twenty-two managers were interviewed representing different kinds of clinics and organisational levels within mental health specialist care in Health Region South—East, Norway (Table 1 for details). In three departments there were two managers from different organisational levels in the same interview, resulting in 19 interviews of 22 persons. For practical purposes, the departments selected are close to the capital region. The choice of managers was criteria-based, with a purpose of obtaining a broad coverage of departments included: in- and out-patient clinics for children and adolescents and for adults as well as patients with a co-morbid condition of drug abuse and mental health problems. The in-patient clinics covered both acute and long-term treatment while the out-patient clinics were regular clinics for mental health treatment. The reason for interviewing managers from all these types of clinics was the chosen focus on patients (adolescents and young adults) representing a complexity of needs and problems receiving treatment and measures from a variety of departments. The managers are 5 males and 17 females in all ages (about 25–65) and with different levels of experience and professional background (doctors, psychologists, an economist, nurses, an occupational therapist and social workers), ranging from team-managers to top-managers. This selection was made so as to provide a broad picture of different organisational contexts and experiences in order to explore and understand the implementation processes. The author asked different managers for an interview out of own knowledge of mental health care, but without knowing most managers personally. No one declined to be interviewed and all found the topic important. The author’s experience with IP, as a clinician and as a lecturer on the topic, had shown a great variability in how IP was introduced and used in different departments and different groups of patients. This provided the background of the project and the chosen group of informants.

### Formalities

All informants signed an informed consent. The project is validated as a quality assurance project by Regional Committee of Ethics of Health Region East. Norwegian Social Science Data Services and The Data Inspectorate have given the necessary approval. The material is anonymous and the names fictive. All interviews were taped and transcribed verbatim.

### Interviews

The in-depth interviews of the chosen managers were, in brief, about their experiences with and reflections on IP as a tool, how they had implemented IP in their organisation and challenges and benefits in use, as well as how they looked at their own role and the staff. All interviews lasted between one and two hours. There was an interview guide, but the questions were evaluated and developed during the period of interviews, according to interesting topics coming up. The total number of interviews was not set in advance, only a decision about having a broad spectre of in- and out-patient clinics (and 20–30 patient cases), to gain width and depth as well as to meet saturation in the material. In this manner the data collection was influenced by Grounded Theory [21].

### Analysis

The analyses were conducted, with a few adjustments done by the author, according to Malteruds Systematic Text Condensation (in short a method of decondensation of text into meaningful units and then to recode these units into categories and further into new descriptions and concepts) [22]. All interviews were consecutively read in a naïve way and notes were made about interesting topics illuminating what was promoting or hindering implementation of IP. Thereafter the interviews were analysed in turn. The broad initial topics were developed further by identifying meaningful units of text from the transcribed material. These units were grouped into sub-categories or categories which evolved during the analytic process. The software program NVivo8 was applied in this part of the analysis. The (sub-) categories were further grouped into those main categories forming the basis of this article: managers’ identification with IP, implementation challenges, practical implementation strategies, leadership style and characteristics of the organisational culture. These categories reflect what the managers emphasised as important dimensions of the implementation process. Since the analysis is data-bound and descriptive and not theory-bound, the categories reflect what the managers related and little revised by the author. At the end of the analytic process the main categories were re-organised and discussed within a frame of implementation theory (Table 2).

## Results

The analytic results illustrate some parts of the managers’ way of carrying out the implementation processes and their reflections on what promoted or hampered the processes. There were considerable differences between work-place situations, organisational levels, how staff related to IP, how the managers interpreted the IP regulations and their own role as well as practical implementation strategies chosen. This nevertheless gives a picture of how IP can be put into action. The evolved categories are thematically organised in line with the presented implementation theory [14, 15], and take into consideration both the organisation and the individual’s readiness and condition to adapt to a change as well as the practical implementation process.

### Organisation

This theme concerns the organisational perspective, where the categories about context and management level, general challenges and culture are assessed to be part of what organisational theory explains as readiness for change, implementation policies and practices and climate.

#### Differentiations in management-level and type of department

Nine managers (upper level) were responsible for several departments or larger units and except for one they did not take part in the daily clinical work. They all believed in IP, but were not directly involved in the implementation process at the same level as those having regular patient contact. Only two from the upper level had directly initiated practical and systematic training/supervision on working with IP. These two worked systematically and over time. Most from upper level were quite eager to keep an eye on the implementation anyway, and one thought she mentioned IP every week when attending staff-meetings: “*I can’t be too grumpy but try to use some humour. The staff knows I’m going to ask about IP. My personal attitude and practice mean a lot.”* A few were at the other end of the scale, and one stated: “*It has not been on my agenda, but it has been a topic for the management team (…) and the team managers work on it as far as I know.*” Three had explicitly delegated the responsibility and practical work with IP to a lower level, but all managers had asked about IP one way or another. Twelve managers were at a lower level. They all believed in IP as a useful tool, but two were somewhat sceptic. Three had initiated a systematic procedure for implementing IP; two had done a little, and eight, nothing at all. Nevertheless, those managers working close to both patients and staff seemed to be role models to another extension than managers from upper level, exemplified by one saying: “*As a role model you have to believe in what you are doing, hold relevant knowledge about IP and being supportive, as well as giving IP a firm foundation in the organisation”.* When looking at the upper level there were no differences between in- and out-patient clinics regarding the initiation of systematic training. At the lower level, seven came from out-patient clinics and of these only one had initiated systematic training/supervision. In the five in-patient clinics two had systematic training/supervision but three did not. Whether there was a child and adolescent clinic or adult clinic made no difference at either level.

#### General implementation challenges

This category reflects on general conditions for implementation in the organisation and how the managers considered the challenges. According to what was told these conditions, often external to the IP itself, made the implementation challenging and frustrating.

##### Too much administration and bureaucracy

To have an increasing amount of administration as part of their daily work, as well as constant new instructions, seemed to be very frustrating for several of the managers. It either took away the focus on implementing IP, or IP was not given priority in the ‘battle of the focus.’ Almost half the managers found the bureaucracy problematic or demotivating, exemplified by the comment*: “The health authorities count plans, without looking for quality. It does not give any motivation, just a feeling of powerlessness and that the bureaucracy is making their own business.*”

##### Lack of resources, power and rights for the patients

This theme was mentioned by most managers, also those most eager to implement IP. “*The case is, there is neither money nor power behind IP, which make it a bit non-sensical”* as one said. They had hoped that IP would give more rights and resources for the patients, like treatment, job/school, housing, etc. Another manager said: “*IP seems to have a frame of consequences that is not true. (…) IP is more like the patient’s yellow pages, it is only an overview. It sounds like a privilege, but the right is only the paper.”*

##### Lack of time

Time was a frequently-mentioned topic in most settings because the managers found IP to be very time-consuming. How they solved this dilemma varied. One, who was not eager for IP, said: “*We don’t have any time to do this; we have to work extra then.”* Most talked about the ever-lasting dilemma of balancing where to give priority, but those managers from in-patient clinics more easily found IP to be part of their daily work. One said: “*This is not supposed to take any extra time since we have to coordinate measures anyway.*”

##### Unclear responsibility

The IP regulation is formulated such that all health personnel have a responsibility to initiate an IP when required, but there is no further description than this which was frustrating and challenging for some managers. Especially those working in in-patient clinics found it problematic to tell if it was their job or not to coordinate an IP, and which generated discussions with representatives from other systems. Some cooperative partners in primary and secondary care had concluded together that “*IP is coordinated from where the patients have their bed*.” In other places this agreement had not been reachable.

#### Characteristics of the organisational culture

According to both organisational theory [14] and diffusion theory [15] the culture or climate in the organisation is essential when it comes to adoption of changes. This has to do both with personal and organisational variables and their interaction. The content of this category is about how the staff cooperates in the implementation process seen from a management perspective. An outpatient clinic manager gave a picture of a kind of organisational cultural obstacle: “*In this system we have a tradition of neglect, more than open resistance,”* while another told: “*I do not think we have an active boycott, but we resist as far as we can. We don’t have to be best in class—is the attitude.*” These two managers, as well as others, told about cultures with a mainly negative attitude against IP, and new procedures in general. They did not say anything about the insecurity often being part of the resistance, or their own position as a manager and a role model accordingly. To cope and to see the purpose seemed to be important. Based on what was told, it took time to gain confidence and have enough knowledge and experience so they could feel they were mastering the new tool. In three in-patient clinics some of the staff were described being anxious about using a computer and did not have routines for documentation. This made the implementation harder. Generally, it seemed that out-patient clinic staff had more training when it came to documentation and being responsible for treatment processes. On the other hand, the in-patient clinic staff had received more training in taking on the coordination work and to take into consideration all aspects of the treatment and need of care for their patients. Some managers really praised their teams for this, as one clearly demonstrated: “*They are never afraid of doing things; I have just said we have to do this, and there have not been any problems*.” One other said: “*People are quite flexible. (…) When I say we have got an instruction, people do as they are asked.*” Three managers told about a positive change in the staff due to age: “*We got some new people in. They were young and more receptive and willing to learn about new things.*” Others talked about general differences in the staff when it came to an enthusiasm for IP, how they took the initiative and how they understood their own position in the IP-process. One out-patient clinic manager summed up her experiences with the staff: “*There has been everything from thinking this is great, to people being angry about more things to do, to some having a guilty conscience for not doing it, and others telling me that I don’t have an idea about how stressful are the workdays they have. There have been all kinds of emotions.*”

### Process

Process refers in this context to what the implementation literature often speak of as the adaptation process. When there is a decision in the organisation about implementing a new tool (like IP) and the organisational readiness for change is clarified, then the process of internal practical work can start.

#### Practical implementation strategies

This category refers to what the managers described they actually did to facilitate the use of IP in daily life; what training, supervision and support was offered. Those managers who were most enthusiastic and believed in IP as a tool were definitely making most of the practical part of the implementation. How they prepared for training and practical work with IP varied considerably. In six organisations they had carried out the training/supervision themselves, and had systematically followed a plan for teaching the staff, demonstrating how IP could be used within meetings and for supervision. Others did it more occasionally or just sent the staff on external courses. A few managers spoke about how they tried to make good routines and procedures for working with IP to make it easier for the team, while others were at the other end such as the one who stated: “*We cannot boast about having any routines at all.*” To have IP as a regular theme at staff-meetings or clinical meetings was the most frequent implementation routine mentioned. Those managers from in-patient clinics were particularly occupied with the practical training; to work with real cases and see how IP could be applied. One said: “*We have started every second week to take all the patients, one by one, and work with their IP. You can’t sit in a room by yourself to work with it; you have to check out your thoughts and comprehensions.”* In out-patient clinics IP was a more general topic at meetings and some leaders made it very simple and gave all the employees a template or an example of how an IP could look like before they talked about it in a few meetings. In two out-patient clinics they had been working systematically together with staff from the primary care, with regular meetings and whole-day courses for all employees. The significance of interactive learning was mentioned by several managers: “*You have to practise, go into real situations to learn.”*

### Individual factors

To separate the individual from the organisational perspective and the adaptation process might be a bit artificial since implementing a tool like IP should be a collective endeavour, but individual variables are essential when it comes to the accomplishment.

#### The managers’ identification with IP

This category concerns how the managers identified with IP in the sense of what they understood IP to be, if they believed in IP to be a useful tool, and how this identification, or lack of it, possibly affected the implementation process. All managers appreciated the intention behind IP and were agreed upon a need for better coordination and collaboration when it came to patients with long-standing complex mental health problems. Four expressed they were sceptical due to the formulation of the guidelines and to their own negative experiences, especially lack of resources and commitment. A few gave the staff only a minimum of information because they did not believe in it as a useful tool in their organisation. Others were ambivalent, mostly due to the general implementation challenges, and therefore seemed to have a lack of motivation when carrying out the implementation. Several managers emphasised the importance of the strengthening of user-participation such as more equality between patients and professionals, increased patient rights and respect for the patients’ goals and wishes. One manager, occupied with user-participation as a right, said: “*User-participation and IP do not necessarily make the patients better, but represent an important democratic right.”* Other benefits mentioned included IP as a way of formalising the work to be done, a tool for cooperation and ‘tidying up’, as well as a way to get a common understanding of the reality, both between patients and service providers and between the service providers themselves.

#### Leadership style and context

This category is built upon the author’s interpretation of the managers’ description of how they have acted in the implementation process and in relation to their staff. Leadership style was not measured. Nor were the staff asked to make an evaluation of their managers, something which makes the category very subjective. The managers differed in how they could be described to be supportive, authoritarian or controlling in the process, or whether they seemed to have a distinct leadership or not. Only a few were explicit on their own positions and how, through their own attitude and practice of leadership, they could encourage the staff to use IP. An experienced manager said: “*If I, as a manager, devaluated IP with saying it’s just another paper, nothing would have happened.*”

##### To check if the work is done

Several managers talked about how they were more or less controlling in the implementation process, but they performed the control in different ways. Four were explicit that to perform control meant a need to check some way, illustrated by a statement such as: “*I have said all patients are going to have an IP, and I will look in the records to control it.*” On the other hand, there were managers who only made IP a topic in their organisation, asked about IP in meetings, or made it part of a supervision setting. Whether a strong control was a suitable way to do it or not is hard to tell without interviewing the staff. Others were clear about their own responsibility: “…*the managers have to hold on to this theme, to give it extra attention until it has been implemented and a matter of course.*”

##### The need to be a supportive manager

To be supportive and caring were explicitly mentioned by four managers, and to keep the staff as part of the implementation process was emphasised by others such as one who stated: “*Of course a manager has to decide, but it is very important to do the ground-work, to make sure all is in and make them see the need and the usefulness of a new tool.*” Another elaborated: “*It is important to point to process, prepare, and try to make it easy, but at the same time be modest…* The importance of motivation and inspiration was also mentioned by a few who described themselves as controlling. “*I try to motivate them one by one and make it pleasurable, at the same time as I can tell: “This you just have to do. I will control it.” Then people start doing it, and the “phase of pain” becomes shorter.*”

## Discussion

The headlines drawn from the presented findings give a picture of what hampers or promotes an implementation process as well as possible benefits and dilemmas. There is a complex interplay between organisational factors, the adaptation process and the influence of individuals when it comes to implementation of IP.

### The results in the light of implementation theory

According to Damschroder et al. [13] implementation barriers arise at multiple levels: the patient level, provider team, organisational level, management and policy level. This is also seen in this study. Several domains, both inner (i.e., leadership, culture) and outer settings (i.e., patient needs, resources) and characteristics of individuals and implementation-processes are recognised as influencing the improvement of delivering care [13, 17, 23]. The meaning of an innovation in terms of its relevance and usefulness has a powerful influence on implementation, and if shared by top-management, team-management, service-users (staff in this study), and other stakeholders, the innovation is more likely to be assimilated [18]. Implementation seemed to be smoother in those organisations or teams where there was a correspondence with existing values, strategies, goals, skills and ways of working. There were significant differences between the clinics when it came to cultures and how the staff related to and showed willingness and flexibility to adopt new tools, as well as how adjustable the structures and procedures in the organisation seemed to be. There were no obvious difference between in- and out-patient clinics or between child- and adolescents’ and adult clinics when it came to the organisations attitude and readiness to change. How the managers perceived the general or external challenges affected how eager they were to give IP priority. Several were disappointed and frustrated according to lack of rights and resources for the patients. They had hoped for more than a legal right for a plan. At the same time they felt the pressure of documentation and priority, which can easily result in ambivalence or neglect in the implementation.

At a policy level, as described in the introduction, the Norwegian health authorities chose a dissemination strategy [7], but without explicit guidelines on how managers should carry out the further implementation in their organisations. From the literature we know that passive dissemination of information is generally ineffective [24] when it comes to implementation of changes. Implementation should contain a systematic strategy to make a long-term change, with ingredients like individual instructions, feedback and reminders as the most effective strategies [19]. Some managers, mainly from lower levels and working close to patients and staff, made IP a regular topic at meetings, and provided supervision as well as arranging systematic training of coordinators. They seemed to have best chance to give IP a permanent place in the clinical work in their organisation. Probably this could reduce the managers need to control if the staff prepared and used their IPs. A few upper level managers told they were mostly asking about IP, not controlling it, due to a position where they found it intrusive to be too controlling. Some managers seemed to be generally quite systematic in their way of working, but none mentioned a long-term plan to implement and keep up the IP work, or related to research or knowledge about implementation.

A Dutch study [25] showed the baseline motivation to implement a tool at the individual level was social support by colleagues, compatibility and perceived relative advantage of the intervention, which is relevant when looking at the findings in this study as well. Other studies have concluded that most implementation barriers are seen within people due to competence, motivation, attitude, personal characteristics like age, experience and self-confidence, learning style and willingness to change [13, 17, 18]. Resource allocation, support and targeted employees seem to be essential. Barriers may arise at multiple levels. A general comment from a manager illustrates the complexity: *“How I would hold on to the implementation would depend on how important I found it, and how important I found it would be partly dependent on my understanding of the topic, and not at least what consequences I could expect when not doing it, or not to give it priority. This, and how useful I found it for the organisation would be decisive for my implementation.”*

Even though most managers did reflect on their own way of controlling the implementation, none of them touched upon their own role in the interaction with the staff. When looking at this study compared to research on successful strategies for change, there is a wide overlap with emphasis on visionary leadership, the manager as a role-model and participatory management style [10, 26, 27] as well as the significance of managerial support in terms of motivation and opportunities [28, 29]. Most mangers talked about a need for motivation and support of the staff, which is important. Motivation determines behaviour and is a predictor of understanding changes, or lack of them [2]. IP was evaluated as ‘a good idea’ from all interviewed managers, but at the same time the tool was forced on people and resulted in some scepticism. To look at determinants of behaviour can be helpful in understanding why managers and staff do as they do in an implementation process. Due to social cognitive theory, behaviour is to a large extent determined by incentives and expectancies. To perceive a need for IP and experience its usefulness makes the implementation a lot easier [18].

### Clinical implications

Transference and relevance of findings is an essential part in research, and this study points out some important topics. Individuals can, of course, put IP into action by themselves, but it is hard to make a new tool succeed that way. If the whole organisation is involved, there is a positive climate for changes, the implementation is systematic and in accordance with relevant knowledge, and there is a match between values, perceived needs and the new tool (IP on this occasion), there is a great chance of succeeding. Managers should take this knowledge into consideration and try to prepare for an implementation with adjustments in all areas. An implementation process needs a long-run priority. To implement and keep up a change is challenging and some estimates indicate that two-thirds of organisational efforts to implement a change fail [13]. Only a relatively few interventions are sustained over time [30] and positive implementation results have often been obtained with levels around 60% [31].

### Limitations and benefits of the study

The study is descriptive and not conclusive and covers only a few informants, but provides a broad picture of different implementation processes as the interviewed managers described these as challenges and possibilities. This picture provides a broad understanding of the processes during implementation [32], but the heterogeneity of informants is a challenge. Different management levels and departments are involved, but they are not easily compatible due to their differences. To acquire a more just picture of the process, especially the role of the manager, some representatives from the staff should have been interviewed about the manager’s leadership style. This would have provided important knowledge due to what we know about the meaning of leadership style, personality and types and bases of power in an organisational change, which an implementation process somehow is. Action research or participating observation could have been another solution. Follow-up interviews of the managers could have checked out the sustainability of their way of working out the implementation process. The author has carried out both the interviews and the analyses, which can be a source of error, even though these have been checked with supervisors. There are both pro and cons in undertaking research in a field known through personal experience. The negative side is primarily the risk of prejudice while the positive side is the possibility of asking relevant questions and validating the relevance of the results.

## Conclusion

What hampers or promotes the implementation of IP is a complex puzzle. IP consists of an interaction of organisational elements, the adaptation process with its practical implementation strategies as well as the involved individuals with their preferences and perceived need of an IP (or not) in their daily work. Whether there is a difference in implementing a tool like IP as opposed to other kind of tools has not been in focus, but to implement an integrated tool like IP is a complex process. The managers have to get IP to function crosswise of administrations, laws and professions, in both primary and secondary health care, which is a complex context. Laws are generally seen as one of the strongest incentives in order to influence behavioural change, but when looking at this study a law and a strategy of dissemination is not fully sufficient in order to implement a tool like IP. The management level is of great importance, but there is a wide range of factors influencing health care delivery. Further research is necessary according to the extensive empirical evidence that organisational factors and the level of implementation affect outcome [31]. This is an argument for planning better implementation strategies than is the case with IP. An understanding of factors underlying IP implementation practice in order to identify which processes should be targeted in implementation interventions as well as to develop an understanding of how the intervention itself work is an essential research focus.

By changing nothing we hang to what we

understand, even if it is the bars of our own jail.

John LeCarre; The Russia House

## Figures and Tables

**Table 1.**
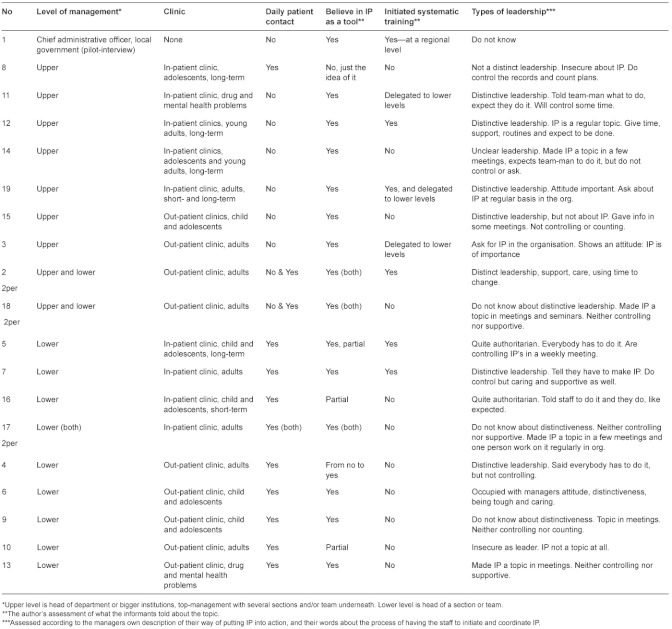
A description of management level (upper, lower), type of institutions (in- and out-patient clinics), belief in IP, systematic implementation training and types of leadership

**Table 2.**
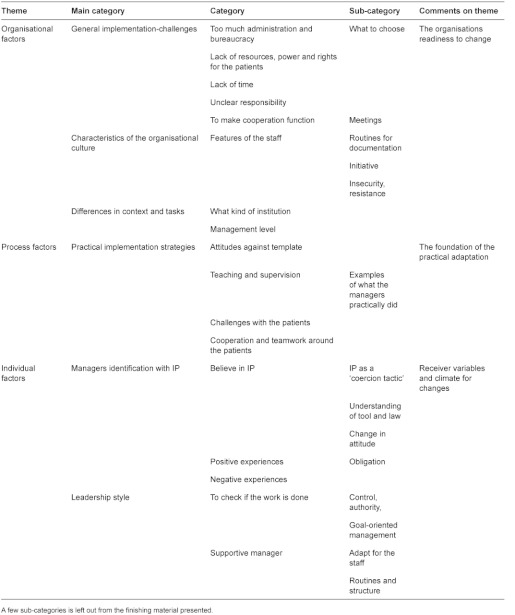
Analytic themes and categories—management interviews
